# Simulations of Promising Indolizidine—*α*6-*β*2 Nicotinic Acetylcholine Receptor Complexes

**DOI:** 10.3390/ijms22157934

**Published:** 2021-07-25

**Authors:** Francis A. Acquah, Matthew Paramel, Adama Kuta, Syed R. Hussaini, David R. Wallace, Blaine H. M. Mooers

**Affiliations:** 1 Department of Biochemistry and Molecular Biology, University of Oklahoma of Health Sciences Center, Oklahoma City, OK 73104, USA; francis-acquah@ouhsc.edu; 2 Department of Chemistry and Biochemistry, The University of Tulsa, Tulsa, OK 74104, USA; mathewparamel@gmail.com (M.P.); adamakuta126@gmail.com (A.K.); syed-hussaini@utulsa.edu (S.R.H.); 3 Department of Pharmacology and Physiology, Oklahoma State University Center for Health Sciences, Tulsa, OK 74107, USA; david.wallace@okstate.edu; 4 Peggy and Charles Stephenson Cancer Center, Oklahoma City, OK 73104, USA; 5 Laboratory of Biomolecular Structure and Function, University of Oklahoma of Health Sciences Center, Oklahoma City, OK 73104, USA

**Keywords:** validation of virtual screening, hetero-oligomer membrane protein modeling, membrane protein-drug complexes, membrane protein dynamics simulations, smoking cessation, lung cancer, antagonists, lead compounds, drug discovery, pore dynamics

## Abstract

Smoking-cessation drugs bind many off-target nicotinic acetylcholine receptors (nAChRs) and cause severe side effects if they are based on nicotine. New drugs that bind only those receptors, such as α6β2* nAChR, implicated in nicotine addiction would avoid the off-target binding. Indolizidine (-)-237D (IND (-)-237D), a bicyclic alkaloid, has been shown to block α6β2* containing nAChRs and functionally inhibit the nicotine-evoked dopamine release. To improve the affinity of indolizidine (-)-237D for α6β2*, we built a library of 2226 analogs. We screened virtually the library against a homology model of α6β2 nAChR that we derived from the recent crystal structure of α4β2 nAChR. We also screened the crystal structure of α4β2 nAChR as a control on specificity. We ranked the compounds based on their predicted free energy of binding. We selected the top eight compounds bound in their best pose and subjected the complexes to 100 ns molecular dynamics simulations to assess the stability of the complexes. All eight analogs formed stable complexes for the duration of the simulations. The results from this work highlight nine distinct analogs of IND (-)-237D with high affinity towards α6β2* nAChR. These leads can be synthesized and tested in in vitro and in vivo studies as lead candidates for drugs to treat nicotine addiction.

## 1. Introduction

The World Health Organization estimates that lung cancer from tobacco smoking causes more than 7 million deaths each year worldwide [1]. Due to high nicotine dependence and adverse withdrawal symptoms, most tobacco smokers who try to quit relapse within the first month of cessation [2]. Nicotine replacement therapy, the most widely used pharmacological intervention for achieving cessation, reduces cravings and withdrawal symptoms in the first eight weeks of treatment but then becomes ineffective [3,4]. Nicotine activates neuronal nicotinic acetylcholine receptors (nAChRs) in the brain. The involvement of nicotinic receptors includes their interactions with the dopaminergic system in substance abuse and neurodegenerative disorders [5,6,7].

Substantial shifts in our understanding of the function of nAChRs have occurred over the last two decades. Initially, all nAChRs were classified as a pentameric ion channel receptor. Early reviews did not mention the α6 subunit [6], stated that the α6 subunit did not form a complex with β subunits, or did so with great difficulty [8]. We now understand that the α6 subtype can complex with the β2 subunit to form the α6β2* nAChR complex and that nAChRs are widely distributed in the brain dependent on their α subunits [7,9]. Normal functioning of nAChRs in the brain involves modulation of transmitter release due to nAChR presynaptic localization [5,8]. Nicotine stimulation of nAChRs has been shown to increase the release of dopamine, norepinephrine, and glutamate [5]. However, not all nAChRs are located on the presynaptic terminal; in some instances, nAChRs have been found on the axon and postsynaptic terminal [5,8]. The activation of these receptors increase the release of dopamine and norepinephrine in the mesolimbic and nigrostriatal areas of the brain. Their release induces rewarding psychoactive effects [10,11]. Thus, nicotinic receptor-based cessation agents offer viable alternatives to nicotine replacement therapy.

Nonetheless, the current nicotinic-receptor-based cessation agents cause adverse side effects, including hypotension and psychiatric symptoms, because they bind to many off-target nAChRs subtypes [12]. More selective drugs would block nicotine-induced dopamine release without off-target effects. We are interested in α6β2* nAChR because it is restricted to the ventral tegmental area (VTA) and nucleus accumbens, regions known for their involvement in reinforcement, sensitization, and locomotion [7,13]. The use of in vivo voltammetry has permitted the application of agents that are selective antagonists for the β2 subunit in the VTA, followed by real-time measurement of in vivo dopamine release in different regions of the limbic system. The addition of dihydro-β-erythroidine (β2 antagonist) significantly reduced dopamine release in the nucleus accumbens, and caudate of mice [14]. Administration of the α6 antagonist, α-conotoxin-MII, significantly reduced dopamine release in the nucleus accumbens, but to a lesser extent in the caudate, suggesting that the α6β2* subtype is the predominate nAChR in the nucleus accumbens. In contrast, the caudate contains other subtypes, such as the α4β2* nAChR [14]. Gotti et al. have continued earlier work to substantiate the importance of the α6β2* subtype in the nucleus accumbens as a critical regulator of dopamine release and a potential mediator of the addictive effects of psychoactive compounds [13]. Using α-conotoxin-MII (α3/α6/β2*-selective) and α-conotoxin-PIA (α6/β2*-selective), they also extended earlier findings by characterizing the nAChR subtype that is found in the caudate, the α4/α6/β2* subtype. The relationship between nAChRs and dopamine release in the nucleus accumbens has led to speculation about the use of compounds that are selective for nAChRs as smoking cessation agents [12]. Recently, interest has increased regarding the potential for α6/β2* subtype-selective indolizidine-type compound development as smoking cessation agents [15,16]. Previous studies found that α6β2* containing nAChRs subtype in the mesolimbic and nigrostriatal areas might play a major role in regulatory dopamine and norepinephrine release [17,18]. Consequently, drugs that target α6β2* containing nAChRs may benefit nicotine addiction treatment.

The bicyclic indolizidines (IND) compounds show promising activity against α6β2* containing nAChRs. For examples, IND (-)-237D has been shown to inhibit nicotine-evoked [^3^H]DA release (IC_50_ = 0.18 nM) in rat striatal slices. Furthermore, good data support IND (-)-237D as a selective inhibitor of α6β2* containing nAChRs [16,19]. These data support our hypothesis that analogs of IND (-)-237D can provide lead compounds that are more potent and selective inhibitors of α6β2* nAChRs.

In this study, we probe our hypothesis *in silico* by characterizing the inhibitor potential of analogs of IND (-)-237D to inhibit α6β2* nAChRs. We used homology modeling, virtual screening, and molecular dynamic simulations to identify high-affinity analogs of IND (-)-237D with antagonism potential. We found eight compounds out of 2226 screened that formed stable complexes in 100 nanosecond molecular dynamics (MD) simulations. We repeated the simulations with the closely related α4β2 nAChR as control on selectivity. However, we did not test all subtytpes, so we did not assess off-targets effects. These results will be of particular interest to medicinal chemists and pharmacologists who are developing potential therapeutics to treat nicotine addiction. The results will also interest neurobiologists seeking more selective inhibitors to better delineate the role of α6β2* nAChR in smoking cessation.

## 2. Results

Below, we present the virtual screening results of 2226 IND (-)-237D analogs and several control compounds against a refined homology model of α6β2 nAChR and the crystal structure of α4β2 nAChR. We selected the analogs with the most favorable docking energies towards the α6β2 but not the α4β2 receptor binding site. Next, we did MD simulations of these complexes to determine if these lead compounds maintained stable interactions with the receptors for the duration of the simulations. Following the simulations, we compared the residence time in the binding pocket and the binding thermodynamics of the ligands. Our analysis showed that the top-ranked IND (-)-237D analogs formed more stable interactions with α6β2* nAChR than nicotine (the native agonist), dihydro-beta-erythroidine (a competitive antagonist), and the parent IND (-)-237D compound *in silico*. Additionally, comparison of the set of ligands against the two receptors showed higher binding affinities towards the α6β2 subtype over the α4β subtype. Because of the large computational effort, we did not extend the analysis to the remaining subtypes, so this study does not assess off-target effects. We also probed the impact of the binding of the analogs on the pore size of the receptor ion channel.

### 2.1. Homology Model of α6β2 Nicotinic Acetylcholine Receptor

**Figure 1:**
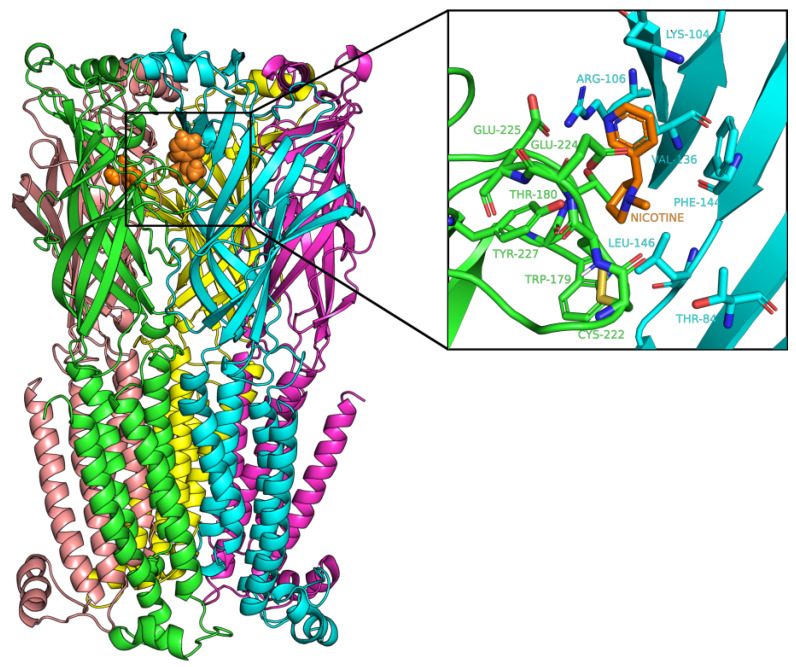
Homology model α6β2 nicotinic receptor. (**Left**) View through the plasma membrane. The alpha helices span the plasma membrane. The two α6 subunits are colored green and yellow, and the three β2 subunits are colored brown, blue, and magenta. Orange van der Waals spheres represent the nicotine. (**Right**) Binding pocket of the receptor showing nicotine as orange sticks and the various binding site residues from α6 chain (green) and β2 chain (blue).

The initial homology model obtained from SWISS-MODEL maintained the canonical structure of nicotinic receptors (i.e., a pentamer with subunit ordering of α6-β2-β2-α6-β2). The subunits had rotational pseudosymmetry around a central ion pore. We modeled the nicotine in the binding site at the α-β interface (Figure 1). Refinement of the atomic coordinates by the YASARA energy minimization server resulted in a model with a low Molprobity clash score (0.16) and few Ramachandran outliers (1.23%) (Table 1). The homology model has 72% overall sequence identity with the crystal structure and 100% identity with the residues within 4 Å around the ligand-binding sites. The results of the structure validation and high sequence identity suggested that the refined homology model was sufficiently accurate for the molecular docking of small molecules.

### 2.2. Library of Indolizidine Analogs

**Figure 2:**
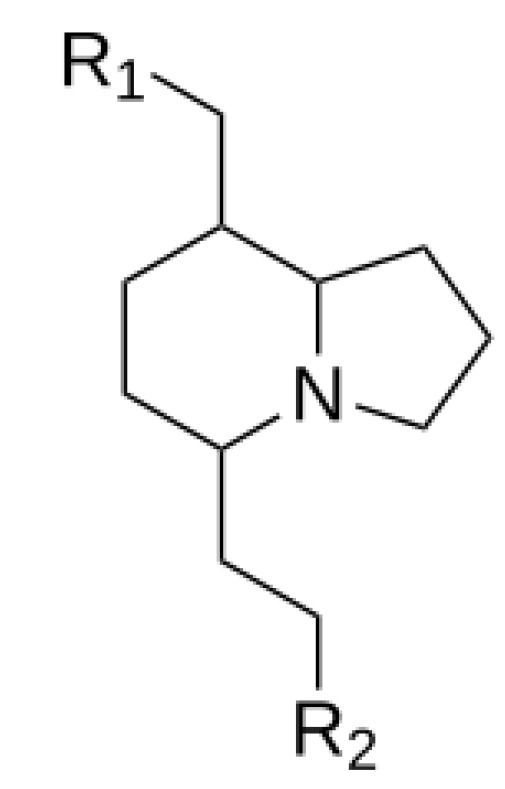
Parent structure of Indolizidine (-)-237D.

We made a library of 5000 analogs of Indolizidine (-)-237D (Figure 2). We then applied filters for favorable pharmacophore properites. These filters reduced the library to 2226 compounds.

### 2.3. Docking Analysis of IND (-)-237D against α6β2 nAChR

**Figure 3:**
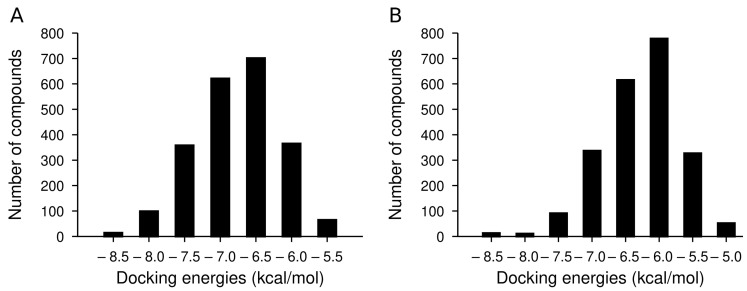
Frequency distribution of docked compounds by their docking energy energies of the 2226 IND analogs against α6β2 (**A**) and α4β2 (**B**) nicotinic receptor. Histogram is divided into bins using a bin width of 0.5 kcal/mol.

**Figure 4:**
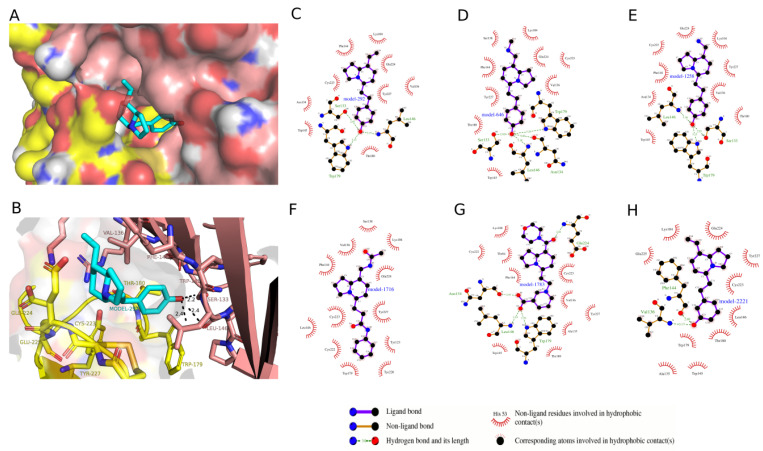
Receptor–analog interactions using PyMOL and LigPlot. (**A**) The docked pose of model-292 in the binding pocket of α6β2 nAChR. Model-292 is shown as a stick model with cyan-colored carbon atoms; the receptor is shown as a molecular surface. (**B**) Model-292 interactions with binding pocket residues. Model-292 is shown as a stick model; α6 chain residues are shown as yellow sticks whiles that of β6 chain residues are shown as brown sticks. (**C**–**H**) LigPlot diagrams of the interactions between top-ranked compounds and the binding site residues of the α6β2 nAChR.

To rank the IND (-)-237D analogs by binding affinity towards α6β2 nAChR for computational studies, we docked the analogs with AutoDock Vina [21]. Docking energies ranged from −8.8 to 5.1 kcal/mol for the α6β2 receptor (Figure 3A). From Figure 3A, compounds in the first and second bins had mean docking energies of −8.44 ± 0.13 kcal/mol and −7.9 ± 0.13 kcal/mol respectively. Welch’s *t*-test comparison of the two bins showed significant difference between the two groups docking energies (*p* value < 0.0001; Welch-corrected t = 13.42; df = 18.75). Compounds in the first bin were considered as the top ranked ligands with most favorable energies at the binding site. The results also revealed that docking energies between these ligands at the two receptor binding site show a higher affinity towards the α6β2 subtype with an average docking energy of −8.5 ± 0.1 kcal/mol and −6.8 ± 0.3 kcal/mol for the α4β2 subtype. Also their docking energies at the α4β2 receptor binding site placed them in the third bin of the frequency distribution (Figure 3B). Thus, these compounds were selected for further comparisons and analysis. The docking scores of the top eight compounds in the first bin, parent IND (-)-237D compounds, and the standard compounds at one of the binding sites of the two receptors are displayed as kcal/mol (Table 2). Top-ranked IND (-)-237D analogs had docking energies two times greater than nicotine and dihydro-beta-erythroidine. Docking analysis showed that the analogs fit snugly into the binding site with the R_1_ substituent pointing out of the binding site and the R_2_ substituent pointing inward (Figure 4A). While the major forces driving the interactions between the analogs and the receptor are hydrophobic, the top-ranked compounds make specific polar contacts with the active site residues (Figure 4C–H). Major interactions made include the R_1_-substituents forming hydrogen-bonds with Glu-224, Cys-223 (β2), Tyr-227 (α6) while R_2_-substituents make hydrogen-bond contacts with Trp-179 (β2), Leu-146, Ser-133, Asn-134, Val-136, Phe-144 (α6) (Figure 4B).

### 2.4. Molecular Dynamic Simulation and Analysis

Next, MD simulations were carried out on each of the top eight ranked receptor-analog complexes to validate the complexes. We checked the stability of the docked complex during the simulation, and we verified the binding affinity results from the docking operations. We extracted the α6β2 and α4β2 nAChRs backbone root-mean-square deviation (RMSD) from the simulation trajectories to check the convergence of the simulation. The RMSD of both receptors in the complexes equilibrated after 20 ns (Figure S1A–F).

**Figure 5:**
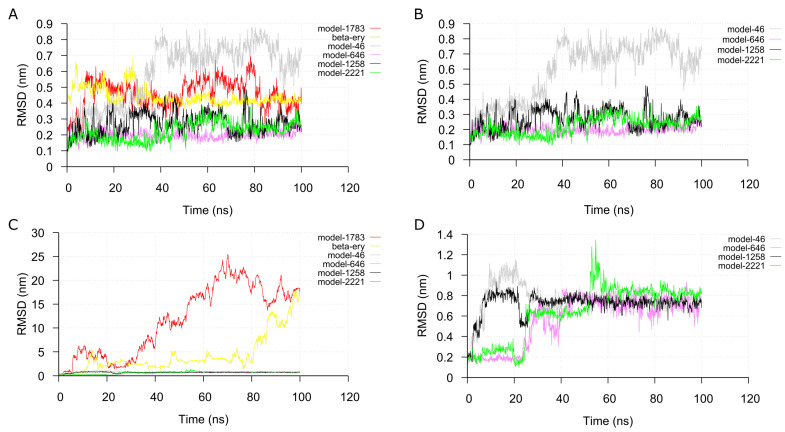
Stability analysis of ligands during simulations. The root-mean-square deviation (RMSD) plot of the compounds in complex with α6β2 (**A**,**B**) or α4β2 (**C**,**D**) receptor with respect to their starting structures as a function of simulation time. [beta-erthry = Dihydro-beta-erythroidine].

Analysis of the RMSD of the analogs in the α6β2 binding sites showed equilibration in the binding pocket for seven out of the top eight analogs simulated (Figure 5C). However, equilibration was only observed for 4 of the analogs at the α4β2 receptor binding sites. Most of the analogs quickly move out of the binding pocket into the bulk solvent after 20 ns resulting in high RMSD values (Figure 5D). Interestingly, at one binding site of the α6β2 receptor, 80% of the analogs that moved out of the pocket and gave high RMSD values were compounds that had low docking energies. These were the lowest-ranked analogs or the classical ligands of the nicotinic receptor. Only one out of the nine top-ranked compounds had high RMSD values. At the two binding sites, high docking energies correlated with low RMSD values during simulation. Conversely, analogs with low docking energies showed high RMSD values, indicating instability and short residence time at the binding sites. Consequently, subsequent analysis focused on the α6β2 nAChR.

**Figure 6:**
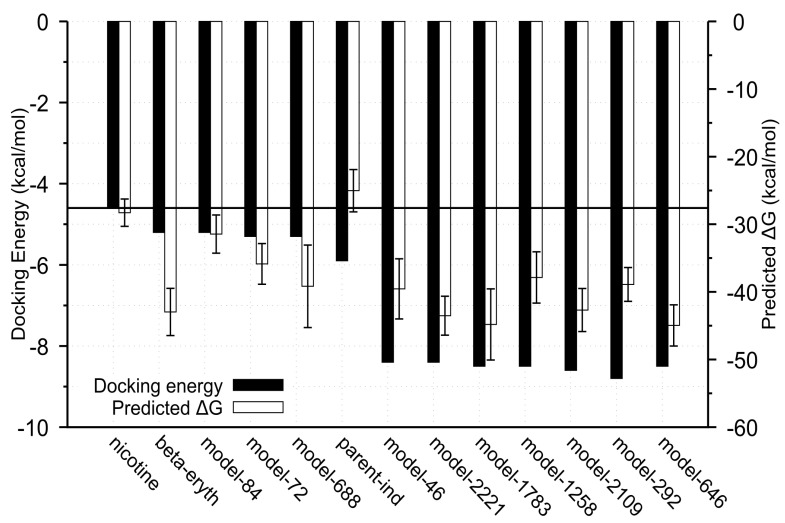
Comparison of docking energy and predicted binding energy from the MMGBSA method. Black horizontal line denotes docking energy of nicotine (classical ligand of the receptor). Model 84, 72 and 688 are the lowest ranked analogs and are added for comparison. [beta-erthry = Dihydro-beta-erythroidine. The predicted binding energies are shown as Mean ± SD; *n* = 1000].

From the simulation trajectories, *gmx_MMPBSA* tool [22,23] was utilized to compute the binding free energy of the MD complexes based on 1000 snapshots taken from the beginning to the end of the simulations using the molecular mechanics-generalized Born surface area (MMGBSA) method. Consistent with previous results, analogs with low average RMSD values also had low binding free energies and vice versa (Figure 6).

**Figure 7:**
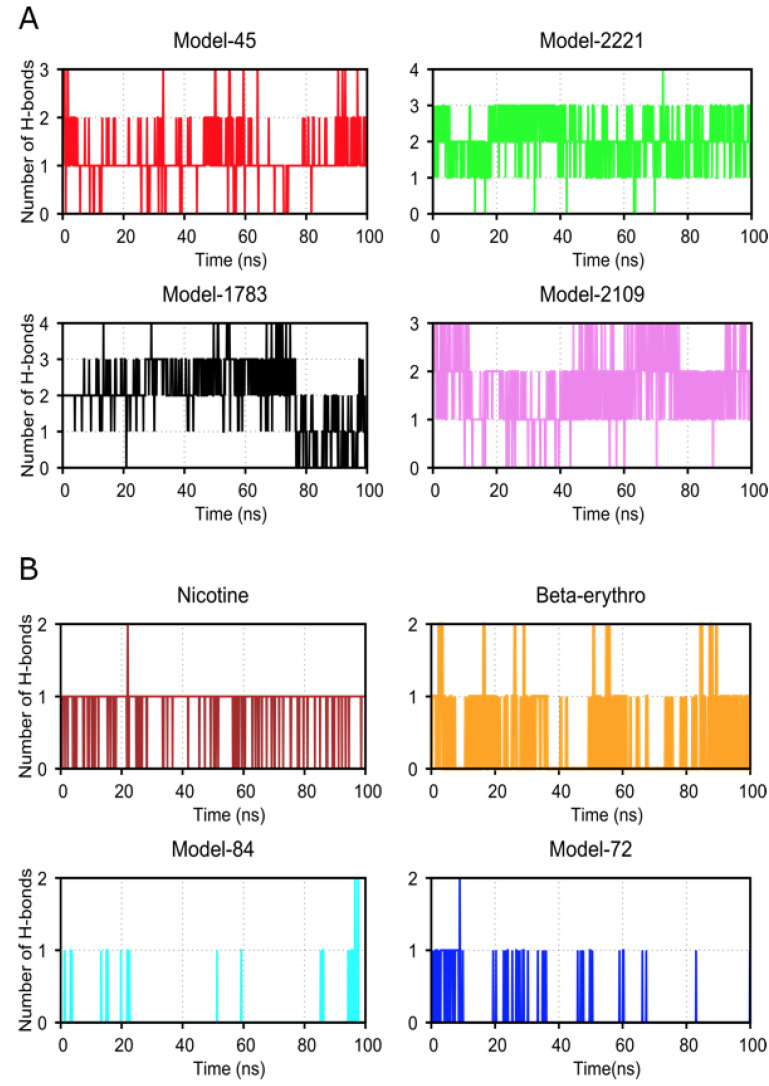
Graph of number of hydrogen bonds formed between receptor and analogs during simulations. (**A**) Number of hydrogen bonds between the four top-ranked compounds and the receptor. (**B**) Number of hydrogen bonds between the four lowest-ranked compounds and the receptor.

To better understand the specific factors that mediate interactions between the top-ranked analogs and the receptor, we analyzed the hydrogen bonds between them by running the gmx hbond utility in GROMACS version 2020.3 [24]. The number of hydrogen bonds for the top four binding analogs ranged from one to four and one to three throughout the simulation. Conversely, the number of hydrogen bonds for the analogs with low docking energy ranged between one and two (Figure 7).

**Figure 8:**
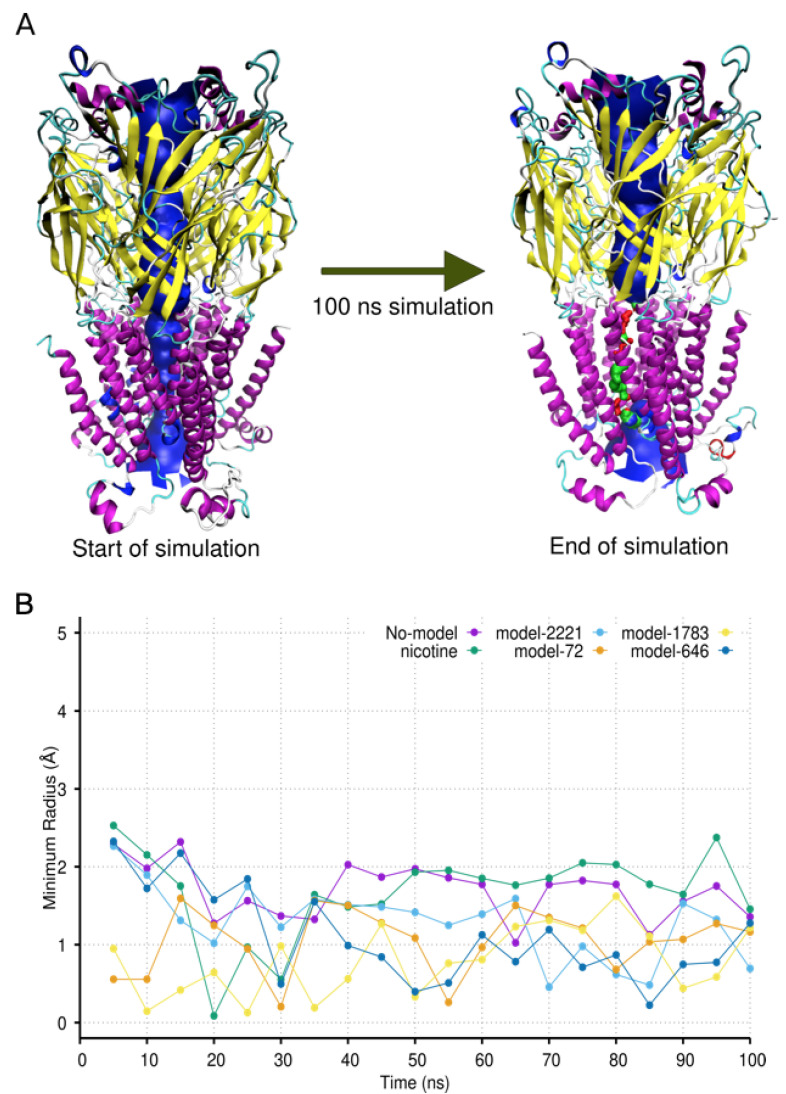
Dynamics of receptor ion channel during simulation. (**A**) Changes in the pore size of the ion channel in the nAChR in complex with model-1783. The pore of the channel is shown in blue, red, or green. Blue denotes an instance where the radius of the pore was large enough for water molecules and cations to pass through. Red denotes where the pore radius was too small for ions and water molecules to pass through the pore. Green denotes a pore radius size sufficient for a single water molecule to pass. (**B**) Fluctuations of the minimum pore radius of the ion channel during the simulation period.

These results support the idea that the lead IND (-)-237D analogs are high-affinity binders of α6β2 nAChR with potential antagonism activity. To ask a more biologically relevant question of how the binding of the compounds affects the dynamics of the ion channel, we analyzed the variation in the pore radius during the simulations. Ideally, for compounds to be considered as agonists or antagonists of an ion channel at the orthosteric site, they should stabilize the protein in a conformation that favors a more open or closed ion channel pore respectively. Analysis of the minimum radius profile along the ion channel of the analog-bound receptors showed a trend towards a more constricted pore at the opening of the transmembrane domain (Figure 8), especially for the for last 50 ns of the simulations (Table 3). Nicotine, the classical agonist for the nicotinic receptor, on the other hand, showed a receptor stabilization towards a more open ion channel.

## 3. Discussion

We aim to develop novel indolizidine compounds that target the α6β2* subtype in the nucleus accumbens and other areas involved in addiction to be used as potential smoking cessation or addiction treatment agents. We know that the areas of significant α6β2* nAChR density are the VTA and nucleus accumbens; both areas are involved in the neurochemical and behavioral changes associated with the addiction process [7,13]. The use of pharmacological agents selective for the various subunits of the α6β2* nAChR has indicated that the α6β2* subtype is involved in regulating dopamine release in the nucleus accumbens following administration of subunit-selective drugs in the VTA [13,14]. The correlation between the localization of the α6β2* nAChR subtype and the data suggest regulation of dopamine release in brain regions associated with addiction has led researchers to find subunit and subtype-selective compounds that are useful for addiction treatment [12].

We have chosen to examine indolizidine compounds’ potential for their selectivity at the α6/β2* subtype as smoking cessation agents [15,16]. To identify high-affinity analogs of indolizidines, we adopted an integrated computational approach that combined protein modeling, virtual screening, and MD simulations to filter promising IND (-)-237D leads from a library of 2226 indolizidines compounds. Traditional chemical screening campaigns after scaffold identification require huge resource expenditure with no guarantee of success [25]. The use of molecular docking that utilizes robust scoring functions represents a resource-efficient strategy in the identification of lead candidates. Our study included the α4β2 subtypes but not the other subtypes. We cannot assess the off-target effects at this time. The lower affinities for α4β2 does suggest that the compounds will have lower affinities for the other subtypes, but this hypothesis remains to be tested.

Additionally, the use of MD simulations as a post-docking step to validate and refine docking results is invaluable in our lead identification pipeline. Coupling MD simulations to docked protein-small molecule complexes has been shown to be very useful in several systems because docking algorithms are imperfect. Aside from screening out binders with bad docking poses manifested as unstable trajectories and high RSMDs with time, MD simulations offer molecular insights such as how binding pocket residues adapt to poses of docked compounds and reveal additional interactions that maintain affinity during the simulation period [26]. An example of this has been seen in how coupling MD with docking was useful in identifying and confirming binding modes of propidium at the peripheral anionic site of the acetylcholinesterase enzyme. In the specific context of nicotinic receptors, MD simulations of dihydro-beta-erythrodione bound to α4β2 receptor revealed structural changes that eventually lead to the closure of the ion pore in the transmembrane domain [27]. In our study, results from the MD simulations of the top docked candidates give further support of those analogs as high-affinity binders as exhibited by low RMSD and low predicted binding free energies towards α6β2 nAChR compared to the α4β2 subtype. It consequently guided their selection as such. It is worth mentioning that the usefulness of this molecular docking and simulations will rest heavily on validation by standard biophysical experiments. This is important as not all computational hits prove to be effective in vitro. Nonetheless, because the set of analogs being screened from the start are derivatives of a scaffold already experimented in vitro and in vivo to be an inhibitor of α6β2* nAChR, our approach is well-suited for quicker lead identification. Additionally, Pivavarchyk et al. have shown that through structure-activity relationship studies, the indolizidine ring is necessary for inhibitory activity [16]. This implies that R_1_ and R_2_ substitutions of the top rank compounds are expected to improve the potency of the parent IND (-)-237D scaffold as a result of high predicted binding energy.

The development of new agents with affinities and potencies better than nicotine and other commercially available compounds is critical to further understanding nAChR involvement in the addiction process. We have seen further characterization of the “TC” compounds as high-affinity nAChR compounds [28,29]. Collectively, there is a lack of α6/β2* subtype-selective agents that could be useful in treating addiction-related or neurodegenerative disorders. This void could be filled by the development of selective α6/β2* drugs using an indolizidine-based compound.

## 4. Materials and Methods

### 4.1. Homology Modeling of α6β2 Nicotinic Acetylcholine Receptor

We made a homology model of the α6β2 nicotinic acetylcholine receptor because no crystal structure of α6β2 was available. The protein sequences with the accession numbers NP_004189.1 (α6) and NP_0007391 (β2) were retrieved from the NCBI Reference Sequences database (RefSeq) [30]. The protein sequences were submitted to the SWISS-MODEL web server to build an initial 3D homology model of α6β2 nAChR using the recently published 3.9 Å resolution crystal structure of α4β2 nAChR (PDB ID: 5KXI) as a template [31,32]. We selected a reliable 3D homology model based on low qualitative model energy analysis (QMEAN) values [33]. Sequence alignment using Clustal Omega version 1.2.4 between the amino acid sequences of the homology model of α6β2 and the template α4β2 was carried out to determine the sequence identity [34].

### 4.2. Structure Assessment, Validation, Refinement of the α6β2 nAChR Homology Model

We accessed the modeled 3D structure of α6β2 in SWISS-MODEL to check the protein geometry. Next, we improved the main chain and side chain stereochemistry by using the YASARA energy minimization server to refine the homology model [35]. We then used MOLPROBITY and PROCHECK to check the stereochemical quality and reliability of the initial and the refined models’ Ramachandran plot and statistics [20,36].

### 4.3. Docking of Indolizidne (-)-237D Derivatives

#### 4.3.1. Modeling of Indolizidne (-)-237D Derivatives

We generated a library of 5,000 compounds using ADMET predictor 8.5 (ADMET Predictor™ (Simulations Plus, Inc. Lancaster, CA, USA; http://www.simulations-plus.com (accessed on 18 July 2018). The library was generated by using the Indolizidine core and modifying the chains containing R_1_ and R_2_. We followed medicinal chemistry guidelines in the selection of R_1_ and R_2_ groups [37].

Next, we used the cApp program to screen for Pan-Assay Interference Compounds (PAINS) [38]. We removed all compounds identified as PAINS from the ADMET (absorption, distribution, metabolism, excretion, toxicity) library. We used another filter in ADMET to detect any drug-like violations. This filter detected ring size > 8, number of rings > 8, rotatable bonds > 10, H-bond donors > 5, H-bond acceptors > 10, Heavy halogen atoms (Cl, Br, I) > 2, aldehydes, sulfonates, hydrazines, acyl hydrazines, hydrazones, dicarbonyls, nitroso, aromatic nitro groups, quinones, alkyl bromides, alkyl chlorides, alkyl iodides, acid halides, sulfonyl chlorides, Michael acceptors, hemiacetals, acetals, hemiketals, ketals, aminals, epoxides, aziridines, thiols, thio carbamates, acid anhydrides, aryl fluorides > 5, isonitriles, acyl ureas, thioureas, isocyanates, thioisocyanates, imines (except amidine or guanine), four valent sulfur connections, 9-aminoacridine, acetate ester > 2, acetylene-heteroatom, activated esters, activated phthalimides, acyl aromatics, acylated enol, acyl hydroxamates, acyl oximes, acyl thiohydroxamates, alkyl sulfite, thiones, ketones, akynylsulfones, allenes, amino esters, rhodanine-like structures, S-S bond containing compounds, dithioesters, thioamides, and phosphinic acids [37,39]. We removed all such compounds from the ADMET list.

We applied another filter called the Central Nervous System Multiparameter Optimization (CNS MPO) to align the compounds with drug-like characteristics further. The CNS MPO scores were calculated according to the literature methods [40]. All structures with a CNS MPO < 4 were excluded from the remainder of the ADMET list of compounds. After applying the above filters, we had 2226 analogs of Indolizidne (-)-237D with distinct substitutions at the R_1_ and R_2_ (Figure 2).

#### 4.3.2. Protein Preparation and Molecular Docking

The refined 3D model of the target protein, α6β2 nAChR was defined as a receptor and prepared for docking by computing the Gasteiger charges and by adding polar hydrogen atoms using AutoDock Tools (ADT) [21]. We saved the prepared file in the pdbqt format [41,42]. We centered the docking grid box (25 × 25 × 25 Å with grid spacing of 0.375 Å) on the classical neurotransmitter binding site for nAChR, which is between the interface of the α6 and β2 chains (76.326, 18.867, and −27.385 in Cartesian space). This box covered the active site residues that were homologous to residues 5 Å around the nicotine in the α4β2 crystal structure. The structures for the above analogs were converted with Open Babel from the Structure Data File (SDF) format to the pdbqt format for use in the docking simulations [43].

Twenty different binding poses were generated for each compound and ranked according to their binding energies during the docking simulations. The pose with the lowest energy of binding was extracted and aligned with the receptor protein for further analysis using PyMOL [44]. Nicotine and dihydro-beta-erythroidine served as control agonists and antagonists, respectively, in the docking simulations and analysis. A similar docking protocol was carried out using the same set of ligands against the crystal structure of α4β2 nAChR. We ran the docking simulations using AutoDock Vina [21] at the Oklahoma Center for Supercomputing in Education and Research (OSCER) at the University of Oklahoma.

### 4.4. Molecular Dynamic Simulation

The coordinates of the top eight docked complexes formed from the molecular docking were used in MD simulations with GROMACS version 2020.3 on the High-Performance Computing Center at Oklahoma State University [24]. The simulated systems were composed of the top eight analogs docked at the two binding sites of the α6β2 nAChR in a membrane modeled as a lipid bilayer. We constructed the protein-compounds-membrane system using the Protein/Membrane System generation option of the membrane builder in CHARMM-GUI [45]. The bilayer was composed of 70% phospholipids and 30% cholesterol molecules. Each layer consisted of 330 lipids and cholesterol molecules. We hydrated the bilayers with water layers covering the ‘extracellular’ and ‘intracellular’ domains of the receptor. An ion concentration of 0.15 M NaCl was used.

The CHARMM36 forcefield with the TIP3P water model were used in the simulations [46,47]. We equilibrated the protein-compounds-membrane complex at constant temperature (310 K) and pressure (1 bar). We held the pressure using a semi-isotropic Parrinello-Rahman barostat with a time constant of 5 ps [48]. The Verlet cutoff scheme was used [49]. Production simulations ran for 100 ns with a time step of 2 fs. The LINCS algorithm was used to constrain bonds containing hydrogen atoms [50]. The electrostatic and van der Waals interactions were calculated with particle-mesh Ewald method during the simulation [51].

## Figures and Tables

**Table 1:** Homology model stereochemistry validation with Molprobity [20].

Parameter	Results (Initial Model)	Results (Refined Model)
Molprobity score	2.49	1.29
Clashscore	5.69	0.16
Ramachandran Favoured	87.36%	92.33%
Ramachandran Outliers	3.04%	1.23%

**Table 2:** Substitution patterns and docking energies of IND (-)-237D analogs with top docking energies.

			Docking Energy (kcal/mol)	
Analog #	R_1_	R_2_	α6β2	α4β2
Model-292	=CH_2_	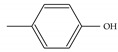	−8.8	−7.0
Model-2109	–CH_3_	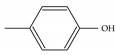	−8.6	−6.9
Model-646	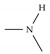	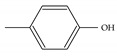	−8.5	−6.9
Model-1258	–NH_2_	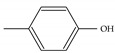	−8.5	−6.8
Model-1716	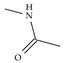	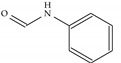	−8.5	−7.2
Model-1783	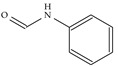	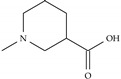	−8.5	−6.3
Model-2221	=CH_2_	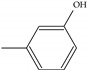	−8.4	−6.7
Model-46	=CH_2_	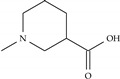	−8.4	−7.1
Indolizidine (-)-237D	–H	–H	−5.9	−5.3
Nicotine	-	-	−4.6	−5.4
Dihydro-beta-erythroidine	-	-	−5.2	−6.5

**Table 3:** Average minimum radius of ion channel pore for last 50 ns of simulation.

Analog	Average Minimum Radius of Pore (Å)
No-model	1.61 ± 0.3
Nicotine	1.88 ± 0.2
Model-688	1.79 ± 0.2
Parent-IND	1.57 ± 0.2
Model-2109	1.56 ± 0.5
Model-46	1.3 ± 0.3
Beta-ery	1.30 ± 0.6
Model-1258	1.26 ± 0.7
Model-84	1.15 ± 0.3
Model-45	1.25 ± 0.3
Model-292	1.11 ± 0.2
Model-2221	1.06 ± 0.4
Model-72	1.05 ± 0.3
Model-1783	0.96 ± 0.4
Model-646	0.78 ± 0.3

## Data Availability

The library of chemical structures of the indole compounds are available from SRH.

## Supplementary Materials

The following are available online at https://www.mdpi.com/article/10.3390/ijms22157934/s1, Figure S1: Stability analysis of ligand-bound receptor during simulations.

## Abbreviations

The following abbreviations are used in this manuscript:

ADMET	absorption, distribution, metabolism, excretion, toxicity
ADT	AutoDock Tools
CNS MPO	Central Nervous System Multiparameter Optimization
COBRE	Center of Biomedical Research Excellence
GPU	Graphical Computing Unit
IND	indolizidines
MD	molecular dynamics
nAChRs	nicotinic acetylcholine receptors
OCAST	Oklahoma Center for the Advancement of Science and Technology
OSCER	Oklahoma Center for Supercomputing in Education and Research
PAINS	Pan-Assay Interference Compounds
PDB	Protein Data Bank
QMEAN	Qualitative Model Energy Analysis
RMSD	root mean square deviation
SDF	Structure Data File
