# Supplementary material for: Simulations of Promising Indolizidine—α6-β2 Nicotinic Acetylcholine Receptor Complexes
Source: Int J Mol Sci. 2021 Jul 25;22(15):7934. doi: 10.3390/ijms22157934 (PMC8347036; doi:10.3390/ijms22157934)
Supplement: Supplementary file 1 [file ijms-22-07934-s001.zip › ijms-1244769-supplementary.pdf]

Supplemental materials

Francis Acquah, Matthew Paramel, Adama Kuta, David R. Wallace, Blaine H. M. Mooers

2021-07-08

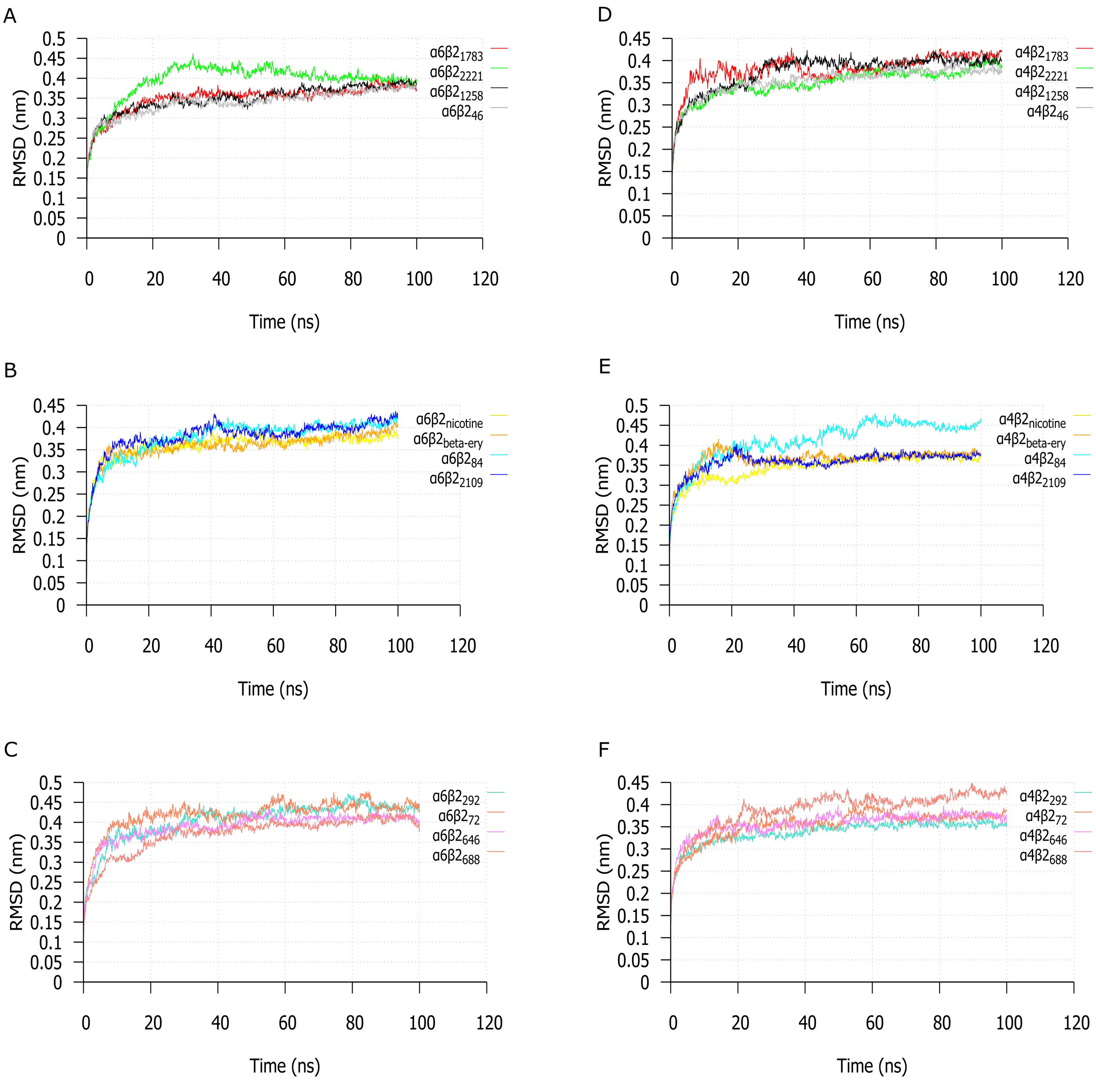

Figure S1: Stability analysis of ligand-bound receptor during simulations. The root-mean-square deviation (RMSD) plot of the backbone atoms of the $\alpha6\beta2$ (**A - C**) and $\alpha4\beta2$ (**D - F**) receptors with different ligands during the 100 ns simulation. [beta-erthry = Dihydro-beta-erythroidine].
